# Genotoxic effects of Roundup Full II® on lymphocytes of *Chaetophractus villosus* (Xenarthra, Mammalia): *In vitro* studies

**DOI:** 10.1371/journal.pone.0182911

**Published:** 2017-08-17

**Authors:** Juan Pablo Luaces, Luis Francisco Rossi, Mónica Gabriela Chirino, Melanie Browne, María Susana Merani, Marta Dolores Mudry

**Affiliations:** 1 Laboratorio de Biología Cromosómica, Facultad de Medicina, Universidad de Buenos Aires, Paraguay, Piso 10 Lab. 6, Ciudad Autónoma de Buenos Aires, Argentina; 2 Consejo Nacional de Investigaciones Científicas y Técnicas, Godoy Cruz, Ciudad Autónoma de Buenos Aires, Argentina; 3 Grupo de Citogenética de Insectos, Depto. Ecología, Genética y Evolución, FCENyN-UBA. Instituto de Ecología, Genética y Evolución de Buenos Aires. IEGEBA-CONICET. Lab. 110. Piso 4 –Pabellón II—Ciudad Universitaria, Intendente Güiraldes, Ciudad Autónoma de Buenos Aires, Argentina; 4 Grupo de Investigación en Biología Evolutiva (GIBE), Depto. Ecología Genética y Evolución, FCEyN-UBA. Instituto de Ecología, Genética y Evolución de Buenos Aires. IEGEBA-CONICET. Labs. 43–46. Piso 4—Pabellón II- Ciudad Universitaria, Intendente Güiraldes, Ciudad Autónoma de Buenos Aires, Argentina; International Nutrition Inc, UNITED STATES

## Abstract

In Argentina, *Chaetophractus villosus* has a wide distribution that overlaps with agricultural areas where soybean is the predominant crop. In such areas the pesticide Roundup Full II^**®**^ (RU) is widely applied. The genotoxic effect of its active ingredient glyphosate (RU is 66.2% glyphosate) on the peripheral blood lymphocytes of *C*. *villosus* was tested over a range of concentrations (280, 420, 560, 1120 μmol/L). Culture medium without glyphosate served as negative control, while medium containing mitomycin C served as positive control. Genetic damage was characterized in terms of the percentage of cells with chromosome aberrations (CA), the mean number of sister chromatid exchanges (SCE) per cell, and the modification of cell proliferation kinetics via the calculation of the replication index. Significant increases (*p* < 0.0001) were seen in the CA frequency and the mean number of SCEs per cell compared to negative controls at all the RU concentrations tested. Chromatid breaks, the only form of CA observed, under the 560 μmol/L RU conditions and in presence of mitomycin C were four to five times more common than at lower concentrations, while no viable cells were seen in the 1120 μmol/L treatment. The mean number of SCEs per cell was significantly higher under the 280 μmol/L RU conditions than the 420 or 560 μmol/L RU conditions; cells cultivated in the presence of MMC also showed significantly more SCEs. All the RU concentrations tested (except in the 1120 μmol/L RU treatment [no viable cells]) induced a significant reduction in the replication index (*p* < 0.0001). The present results confirm the genotoxic effects of RU on *C*. *villosus* lymphocytes *in vitro*, strongly suggesting that exposure to RU could induce DNA damage in *C*. *villosus* wildlife.

## Introduction

The introduction of transgenic crops into Argentina in the late 1990s began an agricultural transformation that included no-tillage systems [1] and the expansion of the country's agricultural frontiers. This transformation, however, required the intensive application of herbicides. One such herbicide, glyphosate [2–3] or *N*-phosphonomethyl glycine, a broad-spectrum herbicide that halts plant growth [4], is usually used in the form of an agrochemical formulation that is more toxic than the herbicide on its own [5]. Commercially available formulations include Roundup Full II^®^ (RU), which is about 66.2% glyphosate; currently this is the most widely used herbicide in Argentina, and indeed the world as [6]. Unfortunately, herbicides and other agrochemicals commonly have unwanted environmental impacts [1, 7]. Indeed, they may cause genetic alterations in wildlife with life-threatening physiological consequences [8, 9]. They may also have teratogenic effects and reduce reproductive success [10]. The characterization of the ecotoxicological damage caused by agrochemical formulations is therefore important.

A number of wild species have been used in the monitoring of environmental contamination [2, 11–14], but only a few studies have employed mammals [15–17]. Studies on the association between exposure to pollutants and changes in the physiology and population dynamics of different mammals, as well as on how pollutants cause habitat loss and disease, and alter natural cycles, are therefore all needed [18]. The present work examines whether *in vitro* exposure to glyphosate causes genetic damage to the peripheral blood lymphocytes of the large hairy armadillo *Chaetophractus villosu*s, a mammalian species with a large geographic range that covers areas of intense herbicide use [1, 19]. We recently described baseline values for the mitotic index (MI), the percentage of cells with chromosome aberrations (CAs), and the mean number of sister chromatid exchanges (SCEs) per cell in *C*. *villosus* captured in pristine areas (i.e., where no agrochemical agents are applied) [20]. Chromosome damage has been used in some ecotoxicological biomonitoring studies as a means of characterizing the genotoxic effects of chemical agents [21, 22], with CAs and SCEs commonly used as cytogenetic endpoints in hazard identification assays [23, 24]. These biomarkers can quickly indicate cytogenetic and DNA damage both *in vivo* and *in vitro* [25–28]. The present results show that exposure to RU causes genetic damage to *C*. *villosus* lymphocytes *in vitro*.

## Materials and methods

### Ethics statement

All animals used in this work were captured in a pristine area of Lucas Monteverde, Province of Buenos Aires, Argentina (35°47’S, 59°99’W). All handling was performed adhering to the Guidelines of the American Society of Mammalogists for the use of wild mammals in research [29]. The experimental protocol was approved by the Committee on the Ethics of Animal Experiments of the University of Morón, Province of Buenos Aires, Argentina (Permit Number: PID15-003/16).

### Blood samples

Peripheral blood samples were obtained from six *C*. *villosus* adults (three males and three females) without anesthesia from the space between the first and second (or the second and third) ring of the tail, using disposable heparinized syringes (Sodic Heparine, PhadaPharma, Buenos Aires, Argentina) equipped with a 21-gauge needle [30]. All animals were immediately released at their capture locations after blood extraction.

### Experimental treatments

Neubauer counting chamber, and blood smears stained with May Gründwald-Giemsa [31], were used to determine the number of lymphocytes per milliliter of blood in each sample, in order to determine the volume of blood required to produce test samples containing 800,000 lymphocytes/mL. Simulating normal body temperature (33–36°C) conditions of *C*. *villosus*, the volumes thus prepared were then cultivated in the dark at 34°C in 10 mL of RPMI 1640 medium containing L-glutamine (Gibco), 10% fetal calf serum (BIOSER), antibiotics (50 U/mL penicillin and 50 mg/mL streptomycin, all from Sigma-Aldrich) and 0.2 ml of 2 mg/mL phytohemagglutinin (PHA-M; final volume 0.02 mg/mL) (Gibco) [32], and in the presence of different concentrations of RU: 280 μmol/L (glyphosate concentration 65 μg/mL), 420 μmol/L (97.5 μg/mL), 560 μmol/L (130 μg/mL) and 1120 μmol/L (260 μg/mL). This range was the same as that used in earlier work with other species [24, 33–34]. CA and SCE frequencies were recorded at 72 h, and the replication index (RI) calculated as shown below. Cultures without glyphosate served as negative controls; positive controls were supplied by cells cultured in the presence of mitomycin C (MMC) (8.97 μmol/L). All experiments were performed after first exposing the cultures to 1 mg/mL 5-bromo-2'-deoxyuridine (BrdU) to assist the viewing of SCE [20].

Colchicine (Sigma-Aldrich, St Louis, MO, USA) at 0.1 μg/ml was added 1 h before cell harvest to arrest all the cell in metaphase; and chromosome preparations made following the standard cytogenetic air-drying method [35]. These slides were then stained following the method of Perry and Wolff [36] to observe CAs and SCEs. Briefly, a few drops of Hoechst 33258 (150 μg/mL) were placed on the slides for 15 min in the dark at room temperature. After washing in distilled water for 3–5 min, they were mounted with 2XSSC (standard saline citrate), and exposed to an ultraviolet light for 2 h at a distance of 15 cm. They were then further incubated in 2XSSC at 60°C for another 2 h before finally staining for 10 min with 3% Giemsa. All experiments were performed in duplicate.

### Measurements of chromosome aberrations, sister chromatid exchanges and cell proliferation kinetics

All slides were coded for blind analysis. CAs were analyzed in cells in metaphase I, and SCEs in cells in metaphase II. CA and SCE values were expressed as means for each individual (taking sex into account); CA values represent the number of cells showing structural chromosome aberrations per 100 metaphases; gaps were excluded. SCEs were analyzed in 60 cells; centromeric exchanges were not included. Cell proliferation kinetics (CPK) were examined (via the RI) taking into account cells that had undergone 1–3 cycles of mitosis (M_1_-M _3_). In the first of these, both chromatids appear dark-stained; in the second, one chromatid of each chromosome is dark-stained; and in the third, chromosomes with both chromatids light-stained or with mixed staining are seen. The RI was established in at least 200 metaphases per animal as follows:

RI = [1 (% of cells in M_1_) + 2 (% of cells in M_2_) + 3 (% of cells in M_3_)]/100 [37]. Metaphases were photographed using a Leitz DMRB microscope and a Leica DFC 300 FX digital camera (Leica Microsystems).

### Statistical analysis

The Mann-Whitney U test was used to compare CA and SCE data for the males and females, and the Student t-test to compare their RI results. Thereafter the data for males and females were analyzed together since no differences were found between the sexes (S1 Table). Differences in CA and SCE frequencies between treated samples and the negative controls (no RU) were analyzed using the Kruskal-Wallis test by ranks (with significance set at *p* < 0.05), followed by the Kruskal-Wallis all-pairwise comparison test for contrasts between treatments (the data were not normally distributed and did not show homoscedasticity). RI values were normally distributed and did show homoscedasticity; comparisons were therefore made using one-way ANOVA (with significance set at *p* < 0.05), plus sequential Bonferroni correction for multiple comparisons. InfoStat software 2015 was used for all statistical analyses.

## Results and discussion

Environmental pollution is major factor in the decline of many species [38, 39]. At present, only a few studies have described the genotoxic effect of agrochemicals on species endemic to Argentina, e.g., on *Caiman latirostris* and *Salvator merianae* [40, 41] as well as on mammals *Mustela vison* and *Mus musculus domesticus* [16, 42]. The present work examines the genotoxic and cytogenetic effects of RU on the armadillo *C*. *villosus*, which has recently been established as a sentinel species [20], via the analysis of CAs, SCEs and the calculation of the RI. Since in the 1120 RU treatment the cells failed to grow, no analyses were made for this concentration.

**Fig 1:**
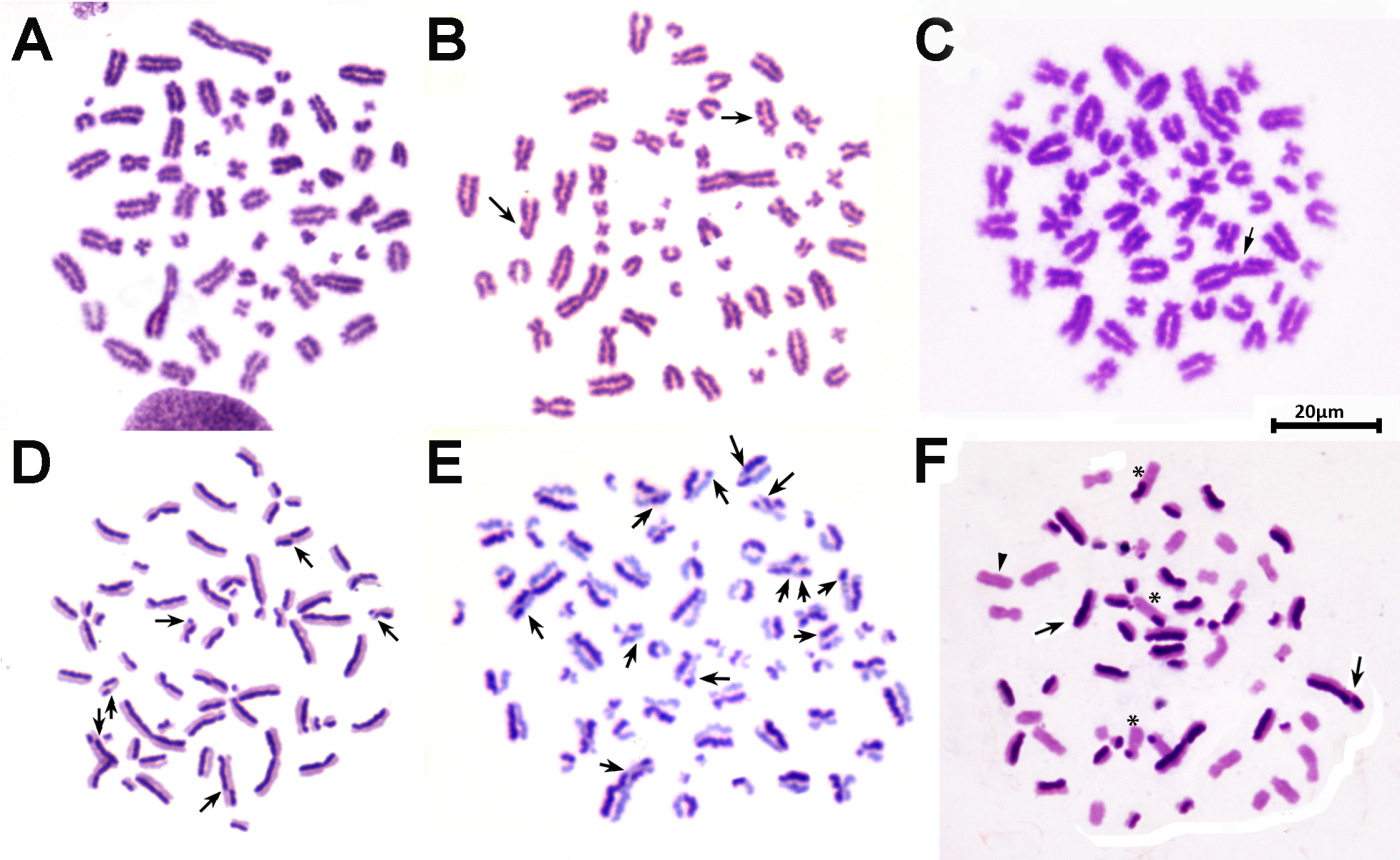
Chromosome aberrations (CA) and sister chromatid exchanges (SCE) in a male *C*. *villosus* (2n = 60, XY). (A) Metaphase (M_1_) from a negative control culture. (B) Metaphase (M_1_) from a lymphocyte culture exposed to MMC as positive control; note the chromatid breaks (arrows). (C) Metaphase (M_1_) from a culture exposed to 560 μmol/L RU showing a chromatid break (arrow). (D) Metaphase (M_2_) from a negative control culture showing the SCE frequency (arrows show the sister chromatid exchanges). (E) Metaphase (M_2_) from a culture exposed to 280 μmol/L RU, with the observed SCE frequency (arrows show the sister chromatid exchanges). (F) Metaphase (M_3_) from a negative control culture having undergone more than two replication cycles: the chromosomes show dark, faint chromatids (arrows; one chromatid also shows an SCE), faintly stained chromatids (arrowheads), and chromosomes with both sister chromatids appearing pale, with a portion that has sister chromatid differentiation (asterisks).

No significant differences were between the sexes with respect to the studied biomarkers: CA: 0.23 ± 0.01and 0.23 ± 0.01 for males and females respectively (*p* > 0.99); SCE: 6.64 ± 0.21 and 6.69 ± 0.16 for males and females respectively (*p* > 0.98); RI: 1.63 ± 0.01 and 1.63 ± 0.02 for males and females respectively (*p* > 0.97). The values obtained for the negative controls are in line with basal values reported in previous work [20] (Table 1; Fig 1A–1C).

**Table 1:** Induction of chromosome aberrations and sister chromatid exchanges by RU in *C*. *villosus* peripheral blood lymphocytes.

RU (μmol/l)	CA (%)***	SCE/cell***	M_1_ (%)	M_2_ (%)	M_3_ (%)	RI***
Negative control	0.23^a^ ± 0.01	6.66 ^e^ ± 0.18	40.15	56.49	3.36	1.63^l^ ± 0.02
280	3.03^a^^b^ ± 0.08	11.02^g^ ± 0.54	64.51	33.37	2.11	1.38^i^ ± 0.01
420	4.01^b^^c^ ± 0.06	8.13^f^ ± 0.30	57.12	37.45	5.44	1.48^k^ ± 0.01
560	14.00^c^^d^ ± 0.11	8.33^f^ ± 0.25	69.30	28.92	1.78	1.33^h^ ± 0.01
1120	—	—	—	—	—	—
MMC, 0.03 μg/ml	16.03^d^ ± 0.09	12.51^g^ ± 0.35	65.19	30.46	4.35	1.39^j^ ± 0.01

Data are presented as means ± standard deviation (n = 12). Abbreviations: *RU*, Roundup; *CA*, chromosome aberrations; *SCE*, sister chromatid exchanges; *RI*, replication index; *MMC*, mitomycin C; *M*_*1*_ *(%)*, percentage of cells in first mitosis; *M*_*2*_ *(%)*, percentage of cells in second mitosis; *M*_*3*_ *(%)*, percentage of cells in third mitosis (for examining CPK via the calculation of RI);—inactive cultures. Means with different superscripts indicate significant differences (***p < 0.0001 for CA, SCE, and RI). Significance determined by ANOVA, followed by Kruskal-Wallis comparisons for CA and SCE, and Bonferroni correction for RI.

^a^ Significantly different from 420 RU, 560 RU, and MMC for CA.

^b^ Significantly different from negative control, 560 RU and MMC for CA.

^c^ Significantly different from negative control, 280 RU, and MMC for CA.

^d^ Significantly different from negative control, 280 RU, and 420 RU for CA.

^e^ Significantly different from negative control versus all experimental conditions for SCE.

^f^ Significantly different from negative control, 280 RU and MMC for SCE.

^g^ Significantly different from negative control, 420 RU, and 560 RU for SCE.

^h-l^ Significantly different among all experimental conditions for RI.

Chromatid breaks were the only kind of structural CA observed, but they well reflect the harmful effects of RU on chromosome replication. The CA frequencies recorded at all RU concentrations (and with MMC) were significantly higher (H = 55.69, *p* < 0.0001) than those recorded for the negative controls (Table 1). In addition, the CA frequencies at 560 μmol/L RU and with the MMC treatment were greater than those recorded for the 280 and 420 μmol/L RU treatments. These findings differ to those made in previous work involving bovine lymphocytes [24], in which the CA frequency recorded at 280 μmol/L RU was close to 1% compared to around 3% in the present *C*. *villosus* cells (3.03 ± 0.08%; Table 1); at 560 μmol/L RU it was just 1% in the bovine lymphocytes compared to around 14% in the present *C*. *villosus* cells (14.00 ± 0.11%, Table 1). It would therefore appear that *C*. *villosus* lymphocytes are more sensitive to the genotoxic effects of RU than are bovine lymphocytes.

The SCE frequencies at all RU concentrations (and in the MMC treatment) differed significantly to those recorded for the negative controls (H = 54.17, *p* < 0.0001) (Table 1; Fig 1D–1F). Strangely, the highest SCE frequencies were seen with the 280 μmol/L RU treatment, indeed they were significantly higher (*p* > 0.05) than with MMC and under the 420 and 560 μmol/L RU conditions. Other authors report increased SCE frequencies in bovine lymphocytes at 420 and 560 μmol/L RU [24]. 1.3 and 1.5 fold increases in SCE frequencies have been reported in human lymphocytes exposed *in vitro* to 438 μmol/L RU and 1446 μmol/L RU respectively [32]. This suggest that the armadillo is more sensitive to RU than other mammalian species [24, 32].

The RI was reduced in all RU treatments (and the MMC treatment) compared to negative controls (F = 1322.16, *p* < 0.0001), with the lowest RI value recorded for the 560 μmol/L RU treatment. As suggested by the SCE results, DNA repair was efficient at 280 μmol/L RU, but poor at 420 μmol/L RU and 560 μmol/L RU; this might be explained by CAs causing delays in the cell cycle. Other authors who examined the effect of RU on CPK in bovine lymphocyte cultures also reported a significant delay in the cell cycle [24]. The absence of living cells in the 1120 μmol/L RU treatment indicates even more damage occurred than at 560 μmol/L RU. M_1_ metaphases were found to be more common at all the RU concentrations tested. This agrees with previous reports in other vertebrate species [5, 24].

*Chetophractus villosus* was recently established as a sentinel species for testing potential genotoxicity [20]. These semi-fossorial armadillos are exposed to different substances in the soils. In Argentina, glyphosate concentrations in field sediments and soils range from 0.5 to 5.0 mg/kg, and in water from 0.10 to 0.70 mg/L [43]; it appears in these media soon application and persist for an unknown period of time [43]. Sentinel species for monitoring its presence are therefore required.

## Conclusions

The present results provide evidence that RU causes chromosomal damage in armadillo lymphocyte cultures, increasing the frequency of CAs and SCEs, as well as delaying the cell cycle. The CA and SCE frequencies is here shown to be of use in genotoxic biomonitoring. It might also be of use in studies with a focus on environmental monitoring, biodiversity protection, and the management of natural environments in this era of high anthropic pressure exerted through increasing agricultural activity.

## Supporting information

S1 Table Effects of genotoxic biomarkers in armadillos’ lymphocyte cultures exposed to different Roundup® (μmol/l) concentrations discriminated by sex. (DOCX)
