# Supplementary material for: Genotoxic effects of Roundup Full II® on lymphocytes of Chaetophractus villosus (Xenarthra, Mammalia): In vitro studies
Source: PLoS One. 2017 Aug 17;12(8):e0182911. doi: 10.1371/journal.pone.0182911 (PMC5560724; doi:10.1371/journal.pone.0182911)
Supplement: S1 Table — (DOCX) [file pone.0182911.s001.docx]

**S1 Table. Effects of genotoxic biomarkers in armadillos’ lymphocyte cultures exposed to different Roundup® (μmol/l) concentrations discriminated by sex**

| Biomarker | Sex | Negative control | RU (μmol/l) | | | | MMC, 0.03 μg/ml | Statistic value | *p-*value |
| --- | --- | --- | --- | --- | --- | --- | --- | --- | --- |
|  |  |  | 280 | 420 | 560 | 1120 |  |  |  |
| CA (%)^a^ | Females | 0.23 ± 0.01 | 3.00 ± 0.09 | 4.03 ± 0.06 | 14.01 ± 0.09 | -- | 1. ± 0.07 | W = 916.0 | 0.9882 |
|  | Males | 0.23 ± 0.01 | 3.03 ± 0.09 | 4.01 ± 0.06 | 13.98 ± 0.14 | -- | 16.04 ± 0.12 |  |  |
| SCE/cell^a^ | Females | 6.69 ± 0.16 | 10.95 ± 0.43 | 8.13 ± 0.25 | 8.40 ± 0.26 | -- | 12.42 ± 0.47 | W = 913.50 | 0.9823 |
|  | Males | 6.64 ± 0.21 | 11.10 ± 0.67 | 8.12 ± 0.36 | 8.27 ± 0.23 | -- | 12.59 ± 0.17 |  |  |
| RI^b^ | Females | 1.63 ± 0.02 | 1.38 ± 0.01 | 1.48 ± 0.01 | 1.32 ± 0.01 | -- | 1.39 ± 0.01 | *t* = 0.004 | 0.9721 |
|  | Males | 1.63 ± 0.01 | 1.38 ± 0.01 | 1.49 ± 0.01 | 1.33 ± 0.01 | -- | 1.39 ± 0.01 |  |  |

Data are presented as mean ± standard deviation for n = 12. *RU* Roundap, *CA* Chromosomal aberrations, *SCE* Sister Chromatid Exchange, *RI* Replication index, *MMC* Mitomycin C, -- Insufficient number of cells.

^a^ Comparisons between sexes were analyzed with the Xilcoxon (Mann-Whitney U) two-tailed test (*p* < 0.05) for CA and SCE.

^b^ Comparisons between sexes were analyzed with the Student’s two-tailed *t*-test (*p* < 0.05) for RI.
